# Electroanalysis and electrophysiological recording of bio‐doped conducting polymer‐modified neural electrodes

**DOI:** 10.1111/nyas.15371

**Published:** 2025-06-05

**Authors:** Alexander R. Harris, Ben J. Allitt, Antonio G. Paolini

**Affiliations:** ^1^ Department of Biomedical Engineering University of Melbourne Melbourne Victoria Australia; ^2^ Higher Education College, Chisholm Institute Dandenong Victoria Australia; ^3^ ISN Psychology, Institute for Social Neuroscience Ivanhoe Victoria Australia; ^4^ School of Psychology and Public Health La Trobe University Bundoora Victoria Australia

**Keywords:** conducting polymer, electroanalysis, electrophysiology, neural implant, neural recording

## Abstract

Electroanalytical methods are used to understand, modify, and control bionic devices. Bionic devices can record or stimulate cells to understand and/or control normal or abnormal biological processes. These devices contain electrodes that transduce electrical current within the electrical circuit into ionic current within a tissue. Despite the similarity between electroanalysis and electrophysiology, there remains a poor understanding of the relationship between the two techniques, including their methodology and theory. This paper investigates the electrochemical and acute electrophysiological recording performance of neural electrodes. A range of behaviors is achieved by modifying electrodes with the conducting polymer poly(3,4‐ethylenedioxythiophene) (PEDOT) doped with chondroitin sulfate, dextran sulfate, or *para*‐toluene sulfonate. The results support previous studies showing that increased electrode area reduced total impedance below the Maxwell–Wagner relaxation frequency and thermal noise while increasing the signal‐to‐noise ratio and neural spike count. The results allowed novel investigation of relative contributions of biological and electrode properties to electrophysiological performance, with increased electrode area having a larger impact on neural population within recording range rather than reducing thermal noise. The utility of measuring electrode impedance for predicting electrophysiological performance is mainly for an indirect measure of electrode area. The results provide insight into noise sources from electrophysiological recordings and limitations in cable theory in neuroscience.

## Graphical abstract

Neural electrodes were modified with poly(3,4‐ethylenedioxythiophene) (PEDOT) doped with chondroitin sulfate or dextran sulfate. Electrochemical and acute electrophysiological recordings were undertaken. The effective electrode area had the strongest correlation with electrophysiological response. The electrode area had a larger impact on the neural population being recorded from, rather than on reducing thermal noise. Cable theory is unrealistic, requiring an extension of electrochemical theory to neural systems.

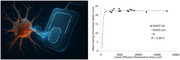

## INTRODUCTION

Electroanalytical methods are used in a range of biological applications that involve the interfacing of electrodes with biological tissue or fluids. These applications include various biosensors and bionic devices for the measurement and control of normal and abnormal biological processes or states.

Bionic devices interface with electrically active cells, including neural tissue and muscle. These cells contain ion channels in their cell membranes, which selectively control the movement of ions in and out of the cell. The ion concentration within a cell is tightly maintained, with ion channels having multiple functions, including controlling cell potential, energetics, development, and signaling processes.^1^ In neurons, signaling naturally starts at the dendrites and travels down to the cell body, axon, and synapse.^2^ This process involves the opening and closing of sodium and potassium ion channels, leading to an increase and then a decrease in cell potential, termed an action potential or spike. When the spike reaches the synapse, neurotransmitters are released, which can interact with a post‐synaptic neuron or cell, altering its behavior.

Bionic devices fall into two categories: neural (or muscle) recording devices and neural (or muscle) stimulators.^3^ Neural recorders measure the change in local potential associated with spikes (nonspecific local variations in ion concentration) and are used to understand and classify electrically active tissue. The output can be used to control prosthetic devices such as a robotic arm^4^ or to provide signals for patient intervention such as predicting the imminent occurrence of an epileptic seizure.^5^ Neural stimulators deliver a charge into tissue to induce or suppress spiking and control tissue function. They can be used to provide sensory cues, such as a cochlear implant for the profoundly deaf;^6^ modify spike timing, such as deep brain stimulation to reduce tremor in Parkinson's disease;^7^ or control tissue function, such as sphincter opening and closing to control bowel function in paralyzed patients.^8^

Typical concerns for bionic devices are their sensitivity to changes in local potential, selectivity for the target tissue (reducing off‐target side effects), biocompatibility (does not induce an immune response), and biostability (does not degrade in the biological environment).^9^ The majority of bionic devices use platinum electrodes, which are highly corrosion‐resistant, conductive, and biocompatible. However, platinum is a very hard material with moderate charge injection capacity, limiting its clinical application to some extent.^10^ There have been ongoing efforts to reduce the size of bionic devices to better target narrow populations of nerves, increasing their selectivity and biocompatibility.^11^ Modification of electrodes, particularly via the application of soft conducting materials or the use of iridium oxide, can enhance the electrical properties of the electrodes, allowing for their miniaturization, and also control biological processes, such as guiding neurite growth to improve device functionality.^12^

This paper uses a range of bio‐doped conducting polymer–modified electrodes to compare electrochemical and electrophysiological responses. Dextran sulfate (DS) and chondroitin sulfate (CS) are novel dopants that impact the hardness, structure, chemical functionality, and conductivity of the electrode surface, which may improve the performance and biocompatibility of the electrode. CS is a glycosaminoglycan, which is a structural component in the body, and is used to treat osteoarthritis. DS is a polysaccharide regularly used as an antithrombotic. Both biopolymers have been used as dopant ions in conducting polymers. CS has been used in polypyrrole (PPy)^13^, ^14^, ^15^, ^16^ and poly(3,4‐ethylenedioxythiophene) [PEDOT],^17^ while DS has been used in PPy.^18^, ^19^, ^20^ These previous studies have demonstrated dopant‐dependent differences in surface roughness and adhered cell viability. As such, the use of these dopants in a neural electrode may provide important cues for controlling cell behavior and may reduce the level of biofouling and glial cell encapsulation, leading to improved long‐term performance.

Although there are increasing numbers of articles describing modified bionic devices with “improved” performance,^21^, ^22^, ^23^, ^24^, ^25^, ^26^ this improvement is typically based on reduced impedance at 1 kHz, increased charge storage capacity measured via cyclic voltammetry, or charge injection capacity measured via chronopotentiometric voltage transients (current pulses). However, there is a very poor understanding of how these electroanalytical methods relate to electrophysiological performance. We have previously published a series of articles developing theory and techniques to investigate the relationship between electrochemical and electrophysiological properties of various electrode materials.^27^, ^28^, ^29^, ^30^, ^31^, ^32^, ^33^, ^34^ This work has demonstrated that electrode impedance is a function of electrode area, that the thermal noise and signal‐to‐noise ratio of neural recording are a function of electrode area, and that various measures of electrode area are possible. In particular, we refer the readers to our previous work discussing the limitations of reporting the impedance at 1 kHz and the need to measure impedance at frequencies below the Maxwell–Wagner relaxation frequency (the cut‐off frequency).^35^, ^36^ ^,^ a However, questions remain regarding the sources of noise in these measurements and how they relate to each other, and the relative contributions of biological and electrode properties to electrophysiological performance. The results obtained in this article provide new insights into these issues and guide future approaches for developing next‐generation bionic devices.

## MATERIALS AND METHODS

The methods are largely the same as previously reported,^35^ but are replicated here for the reader's convenience.

### Materials and electrode coating

3,4‐Ethylenedioxythiophene (EDOT), chondroitin sulfate A sodium salt from bovine trachea (MW ∼31,000), dextran sulfate sodium salt (average MW > 500,000), sodium *para*‐toluene sulfonate (Na_2_pTs), hexaammineruthenium(III) chloride (Ru(NH_3_)_6_ Cl_3_) (Sigma‐Aldrich), and 99.0% di‐sodium phosphate (Fluka) were used as received. Polymer coatings were deposited on 4‐shank, 32‐electrode (8 electrodes per shank), 413 µm^2^‐nominal geometric area platinum electrodes with 200 µm pitch (Neuronexus Technologies–A4×8‐5mm‐200‐200‐413). Conducting polymer coatings with different dopants were electrochemically deposited onto individual microelectrodes via a potentiostat (CH660D, CH Instruments) from mixed solutions containing 10 mM EDOT and 0.1 M Na_2_pTs or 2 mg mL^−1^ CS or DS in deionized water. Potentiostatic growth was performed in a three‐electrode configuration using one microelectrode as the working electrode, Ag/AgCl (3 M NaCl) as reference electrode and Pt mesh as counter electrode. Solutions were degassed for 30 min with nitrogen before depositing the electrode coatings. All polymers were deposited at 1 V versus Ag/AgCl. PEDOT‐CS and PEDOT‐DS were deposited for four different times (15, 30, 45, or 60 s), PEDOT‐pTs was deposited for 45 s as recommended in our previous paper.^27^ Two probes were coated with PEDOT‐CS and two with PEDOT‐DS, four electrode sites coated at each deposition time in a staggered array as previously described,^27^ leaving 12 uncoated platinum electrodes and four PEDOT‐pTs coated electrodes as controls.

**FIGURE 1:**
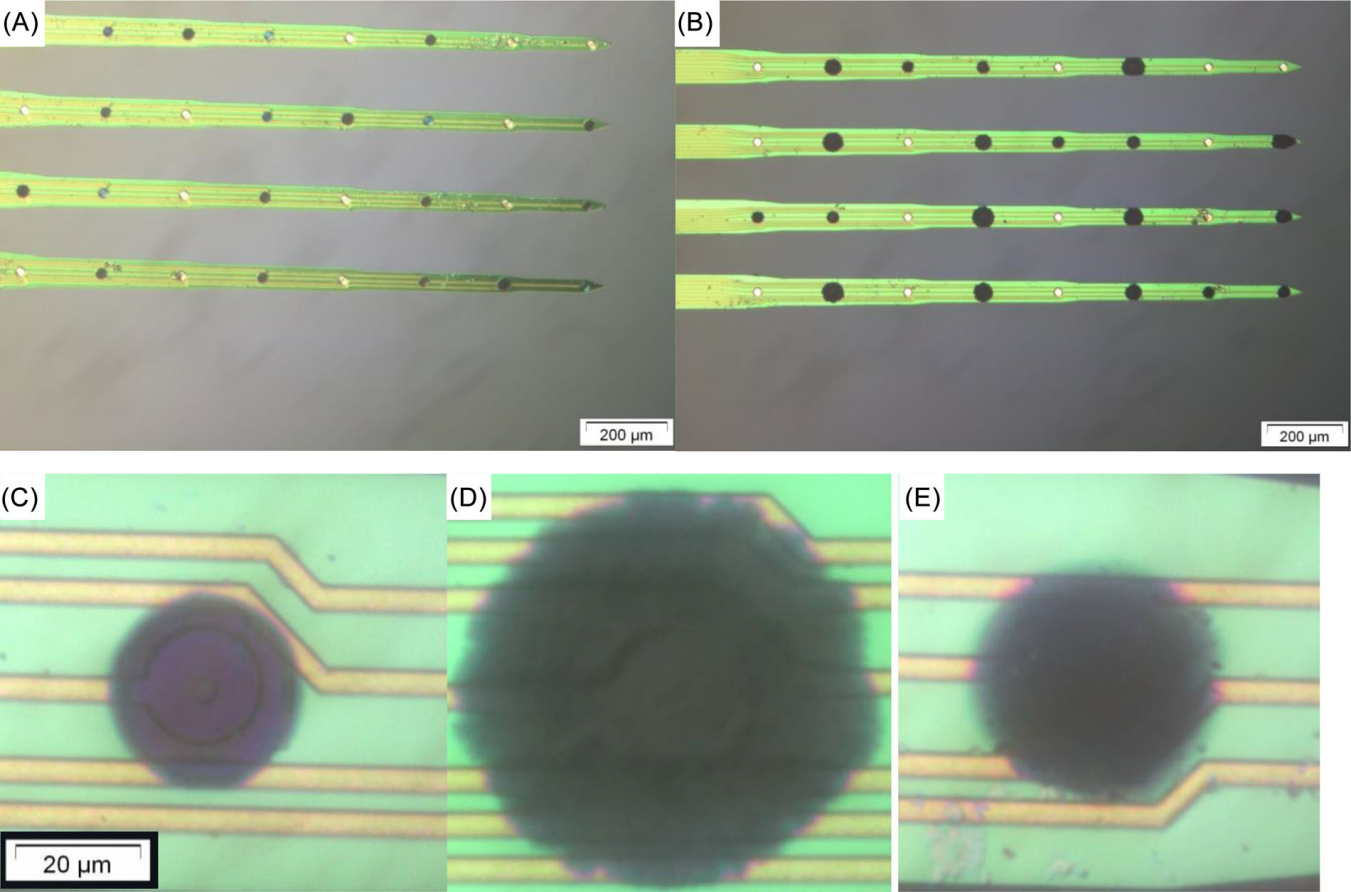
Visible light microscopy of (A) PEDOT‐CS‐ and (B) PEDOT‐DS‐modified probes, and higher magnification images of 45‐s deposition of (C) PEDOT‐CS, (D) PEDOT‐DS, and (E) PEDOT‐pTs.

Electrodes were imaged using a BX61 optical microscope (Olympus), and the geometric area was measured with ImageJ (Figure 1). Electrochemical analysis was undertaken in 0.3 M phosphate buffer in deionized water (as was recommended by the electrode manufacturer), and the electroactive areas were measured by the addition of 5 mM Ru(NH_3_)_6_^3+^. Test solutions were not degassed. A CHI660B potentiostat with CHI684 multiplexer (CH Instruments) was used to perform cyclic voltammetry at each of the individually addressable working electrode sites. A three‐electrode configuration was used with an Ag/AgCl (3 M KCl) reference and Pt mesh counter electrode. Cyclic voltammetry was performed over the “safe” water window range of 0.8 to −0.8 V versus Ag/AgCl at a scan rate of 100 mV s^−1^. Electroactive area measurements were undertaken over a range of 0 to −0.5 V, varying the scan rate from 10 mV s^−1^ to 1 V s^−1^. Electrochemical impedance spectroscopy (EIS) was performed at 0 V with a 10 mV amplitude over a frequency range of 10–100,000 Hz to compare with previous data on conducting polymer–modified neural electrodes. Equivalent circuit fitting of the EIS data was performed with ZView.

### In vivo testing

Experimental procedures were performed in a sound‐attenuating Faraday cage on an antivibration table. Hooded Wistar rats (four in total) weighing over 200 g were anesthetized with urethane (20% v/v in distilled water, 1.3 g/kg i.p., Sigma‐Aldrich). The animal was secured in a stereotaxic frame (David Kopf Instruments) fitted with a hollow ear bar in the left ear. Animal temperature was monitored continuously via a rectal probe and maintained at 37.5°C using an ATC1000 DC temperature controller (World Precision Instruments). A craniectomy was performed to access the right inferior colliculus (IC). An Ag/AgCl wire reference electrode wrapped in saline‐saturated cotton wool was placed into the dorsal region of the animals’ neck. The multichannel polymer electrode was then inserted at a 19° rostro‐caudal angle with reference to Lambda using stereotaxic coordinates and a rat brain atlas^37^ approximately 2 mm into the brain, toward the IC. White noise bursts were generated by an RX6 multifunction processor and a PA5 programmable attenuator (Tucker‐Davis Technologies) controlled by custom software developed in OpenEx. Sound was delivered through the left ear bar using an EC1 electrostatic speaker driven using an ED1 electrostatic speaker driver (Tucker‐Davis Technologies). Prior to use, the speaker was calibrated by attachment of the sound generation system to one end of the ear bar with a one‐eighth‐inch 4138‐A‐015 microphone and amplifier unit and 2829 4‐Channel Microphone Power Supply (Brüel and Kjær) coupled to the other end using a 3 mm long rigid plastic tube to mimic the rat's ear canal. The electrode was then advanced into the IC using a motorized microdrive (Sutter Instruments), while monitoring the neural response via a PZ2 high impedance amplifier and RZ2 bioamp processor (Tucker‐Davis Technologies) with band‐pass filtering (300–5000 Hz), until roughly the bottom three electrodes on each shank displayed acoustically driven activity.

An acoustic stimulation protocol of 300 repetitions of 50 ms white noise bursts (rise–fall time 10 ms, Gaussian distributed noise, 1–44 kHz) at a 1‐s repetition rate was then delivered at 70 dB sound pressure level, while recording the multiunit activity at each electrode (acquired at a sampling rate of 24.4 kHz). On completion of the acoustic stimulation protocol, the probe was advanced ∼200 µm into the IC so that each electrode was in approximately the same position as the more distal electrode from the first measurement. The acoustic stimulation was then repeated, and the probe advanced in 200‐µm steps until all of the electrodes had recorded acoustically evoked activity. The probe was then retracted in 200‐µm steps using the same acoustic stimulation protocol to determine the reproducibility of the measurements and potential damage caused by the probe insertion. After in vivo recording, the electrodes were carefully retracted from the animal and gently rinsed with deionized water before storing. All animal procedures were in accordance with the *Australian code for the care and use of animals for scientific purposes* and were approved by the RMIT University Animal Ethics Committee (AEC Number 1315). A comprehensive description of all procedures and electrodes was published previously.^28^

### Data analysis

Data from acoustically evoked responses were imported into MATLAB for offline analysis. A Fourier transform of the complete 300‐s noise pulse train was performed. A bandpass filter of 300–5000 Hz was applied for measuring multiunit activity. For each electrode site, the average of the bandpass filtered root mean square (RMS) measured during acoustic stimulation (RMS_stim_) for the complete 50 ms stimulation period was averaged from the 300 repetitions at one electrode depth. The average of the bandpass filtered RMS outside the acoustic stimulation period (RMS_bkgd_) was only performed over the last 300 ms of the 950 ms time and averaged from the 300 repetitions at one electrode depth to eliminate artifacts from neuron refractory periods. The signal‐to‐noise ratio (SNR) was calculated from (RMS_stim_ / RMS_bkgd_) with the SNR classification taken from Ref. 38 (where low SNR: <3.5, medium SNR: 3.5–4.0, and good SNR: >4.0). A spike was measured where the recorded potential was >4.2 × S.D. of the RMS from the previous 1 s with an exponential weighting of signal for recency. The “during” and “outside” acoustic stimulation spike count was then performed over the same time periods as above. The spike count difference was calculated from (spike count during stimulation) − (spike count outside stimulation). Each electrode site was classified as “in” the IC when acoustically driven neural activity induced a spike count difference greater than 45% of the maximum recorded spike count difference over the whole experiment. Data for each electrode site was averaged across all electrode depths “in” the IC to reduce error due to variations in biological noise (the number of recordable neurons in the vicinity of the electrodes). To determine the impact of electrochemical behavior on electrophysiological performance, each electrode site was analyzed and plotted individually to determine correlation coefficients.

## RESULTS AND DISCUSSION

### Microscopy and electroanalysis

The electrodeposited, conducting polymer–modified electrodes were imaged by optical microscopy (see Refs. 30 and 33 for detailed analysis). The uncoated platinum electrodes were bright silver, while the conducting polymers were a dark blue (Figure 1). The geometric area of the conducting polymer deposited increased with deposition time, with PEDOT‐DS appearing nodular and rough, while PEDOT‐CS and PEDOT‐pTs were smoother and more uniform. At longer deposition times, the PEDOT‐DS grew to the edge of the electrode shanks, which was more pronounced at the thinner shank tips. Previous publications have shown the correlations between electrode area, deposition charge, charge density, and charge storage capacity (CSC, determined by integrating the cyclic voltammetry/time curve) were impacted by the conducting polymer touching the shank edge.^29^, ^30^ However, conducting polymers touching the shank edge had no impact on the impedance^31^, ^32^ and were not identified as outliers in any of the electrophysiological responses reported in this article.

**FIGURE 2:**
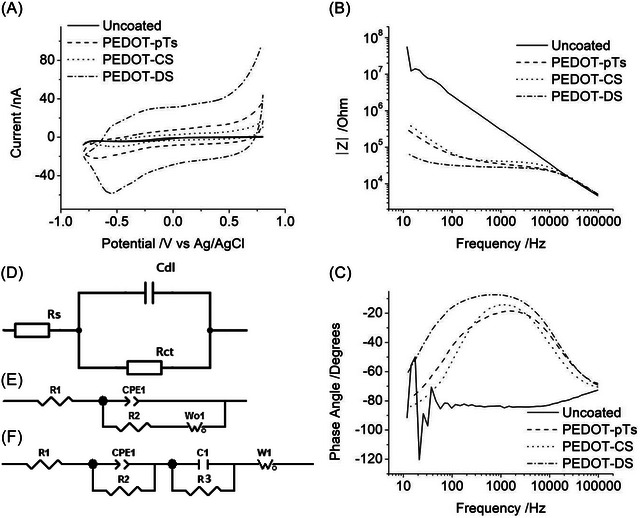
(A) Typical cyclic voltammetry at 100 mV s^−1^, and electrochemical impedance spectroscopy in 0.3 M Na_2_HPO_4_ of (B) an uncoated electrode and (C) electrodes coated at 45‐s deposition of PEDOT‐pTs, PEDOT‐CS, or PEDOT‐DS. Equivalent circuits are fitted to (D) PEDOT‐pTs, (E) PEDOT‐CS, and (F) PEDOT‐DS.

**FIGURE 3:**
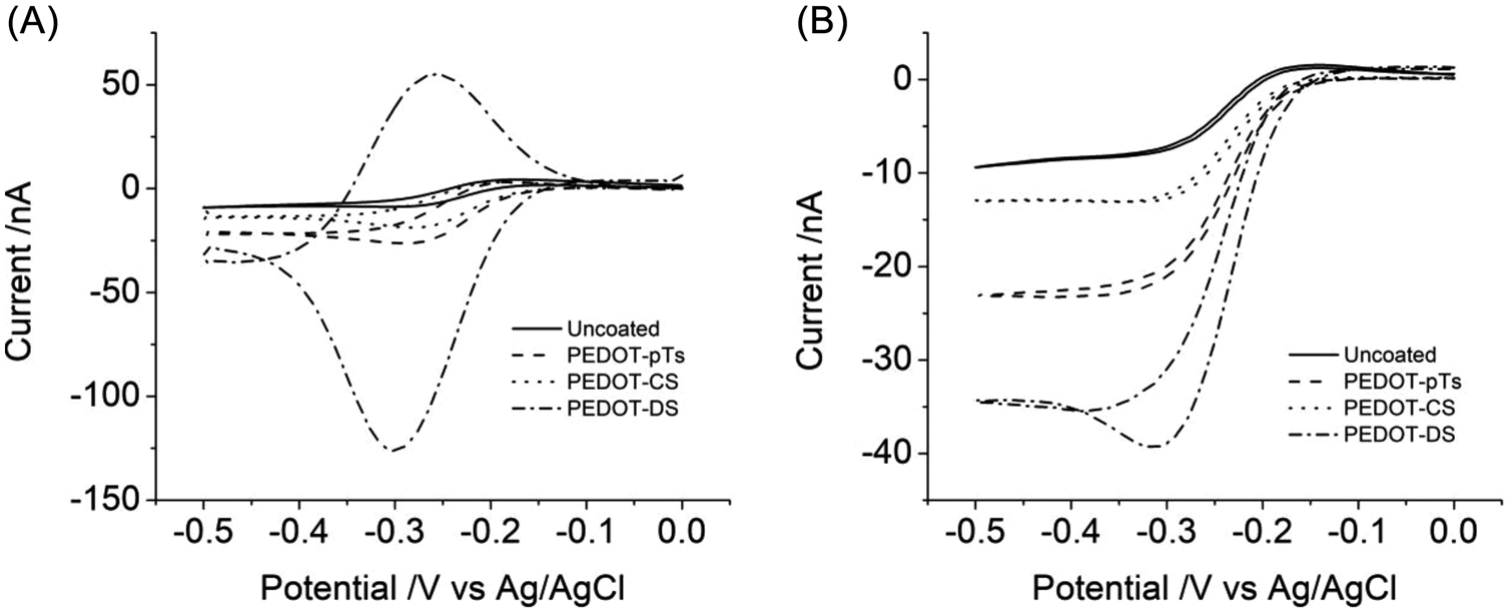
Typical background subtracted cyclic voltammetry of 5 mM Ru(NH_3_)_6_^3+^ in 0.3 M Na_2_HPO_4_ on uncoated electrode; and electrodes coated at 45‐s deposition of PEDOT‐pTs, PEDOT‐CS or PEDOT‐DS at (A) 200 mV s^−1^ and (B) 10 mV s^−1^.

Electroanalysis was previously reported for each doped conducting polymer in nondegassed 0.3 M Na_2_HPO_4_ (Figure 2).^27^, ^29^, ^30^, ^31^, ^32^, ^33^, ^34^ The electrochemical mechanisms occurring at each of the electrode materials were thoroughly discussed in the previous publications; only the relationship between the electrochemical and electrophysiological properties is detailed in the present paper. Figures 2 and 3 are included here to summarize the previous work. Briefly, platinum displayed a relatively small capacitance current with reduction processes due to the reduction of oxygen, platinum oxide, and hydrogen adsorption and oxidation current from hydrogen desorption, platinum oxide formation, and phosphate adsorption (Figure 2A).^10^ PEDOT‐pTs and PEDOT‐CS had a slightly larger capacitance current with a small reduction process below −0.5 V.^33^ PEDOT‐DS had a substantially larger capacitance current and a more pronounced reduction process around −0.5 V.^30^ The current magnitude was previously shown to increase with increasing deposition time for each conducting polymer.^30^, ^33^

EIS was undertaken at 0 V rather than the open circuit potential to enable comparison of each electrode coating (Figure 2B,C). Platinum had a decreasing trend of total impedance versus frequency and a phase angle close to −90°, typical of a series resistor–capacitor (RC) equivalent circuit. All doped PEDOT‐modified electrodes had a plateau in the total impedance with the phase angle approaching 0° between 100 and 10,000 Hz. While the total impedance at 1 kHz was relatively stable for each doped conducting polymer with increased deposition times, the impedance at low frequencies decreased substantially.^32^, ^34^ The transition in the phase angle from a capacitive response (close to −90°) toward a resistive behavior (of 0°) is a relative function of the charge relaxation time (dielectric constant) of the electrode double layer and the bulk electrolyte solution. The electrode double layer dominates the EIS behavior at low frequencies, while the electrolyte dominates the behavior at high frequencies. The transition between these two regions (at −45°) is termed the Maxwell–Wagner relaxation frequency (or cut‐off frequency). With a 45‐s deposition time, the Maxwell–Wagner relaxation frequency increased with PEDOT‐DS < PEDOT‐pTs < PEDOT‐CS < Uncoated. However, the exact measurement of the Maxwell–Wagner relaxation frequency is difficult, requiring the use of a Luggin capillary reference electrode; as a relative function of the dielectric constant at the electrode–solution interface, is dependent on experimental conditions, limiting the relevance of in vitro measurements in understanding in vivo behavior; and ultimately provides information which is of limited utility in assessing bionic electrodes. Satisfactory equivalent circuit fitting of PEDOT‐pTs could be achieved with a simplified Randles circuit (Figure 2D),^31^ PEDOT‐CS displayed a second time constant requiring the inclusion of a Warburg element (Figure 2E),^34^ while PEDOT‐DS required the addition of a third‐time constant (Figure 2F).^32^ Further details on the equivalent circuit fitting can be seen in previous publications.^32^, ^34^

The effective electrode area was assessed by a reduction of 5 mM Ru(NH_3_)_6_^3+^ (Figure 3).^29^ At fast voltammetric scan rates, the Ru(NH_3_)_6_^3+^ diffusion profile was linear, enabling the application of the Randles–Sevcik equation to measure the electroactive electrode area, including surface roughness. In this case, the peak heights (*i*_p_) can be used to calculate a linear diffusion effective area (*A*) according to

(1) $$i_{p}=\left(2.69\times 10^{5}\right)n^{3/2}AD^{1/2}c\upsilon^{1/2},$$

where *n* is the number of electrons transferred, *D* is the diffusion coefficient (9.0 × 10^−6^ cm^2^ s^−1^),^39^ *c* is the concentration, and υ is the scan rate. At slow scan rates, a steady‐state voltammetric response associated with a radial diffusion profile could be achieved on bare platinum and PEDOT‐pTs‐ and PEDOT‐CS‐modified electrodes, but not on the larger PEDOT‐DS‐modified electrodes. The limiting current (*i*_ss_) at a disc electrode has the form

(2) $$i_{\mathrm{ss}}=4nFDcr,$$

where *F* is the Faraday constant and *r* is the electrode radius. For both measurement techniques, the electrode area increased with deposition time, and at a 45‐s deposition time, the electrode areas increased from uncoated Pt<PEDOT‐CS<PEDOT‐pTs<PEDOT‐DS. Further discussion on the charge density (CSC divided by electrode area) of each electrode material can be found in previous publications.^29^, ^30^, ^33^

### Electrophysiology

Following electroanalysis, the electrodes were implanted into rat animal models for acute electrophysiological recording. The electrophysiological response of each individually addressable electrode was obtained versus an Ag/AgCl wire reference electrode wrapped in saline‐saturated cotton wool. The change in reference electrode between in vitro and in vivo experiments shifted the DC potential of the working electrodes.^40^ The implanted electrodes were also exposed to a range of biomolecules, leading to protein adsorption and a further shift in the electrode DC potentials (due to the formation of a Donnan potential).^41^, ^42^ However, for electrophysiological recordings, the electrode potential is high‐pass filtered, removing the impact of both of these changes in DC potential on the analyzed data.

**FIGURE 4:**
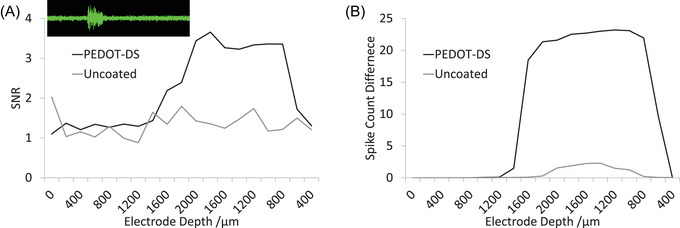
(A) Typical signal‐to‐noise ratio (SNR) and (B) spike count difference of a PEDOT‐DS and an uncoated electrode during insertion and withdrawal into the inferior colliculus (IC). Inset of Panel A: Typical streaming data showing RMS_bkgd_ of ∼10 µV with increased RMS_stim_ during acoustic stimulation from a 50 ms white noise burst.

The streaming data (inset of Figure 4A) of the high‐pass filtered electrophysiological recording appeared as a white noise on all of the electrodes^28^ with an RMS_bkgd_ of approximately 10–20 µV (Table 1). RMS_bkgd_ arises from spontaneous biological activity and thermal or electrochemical potential noise ($V_{rms}^{th}$) due to fluctuations of charge carriers (electrons and ions) and associated voltage fluctuation. During acoustic stimulation, biological activity increases, raising the RMS level, with some electrodes having an RMS_stim_ of over 70 µV. This resulted in the SNR varying from 1 to over 7, depending on the electrode coating and the position within the IC. The total spike count was also measured, which was always <1 outside the acoustic stimulation period and increased to a maximum of ∼25 spikes within the 50 ms stimulation period (∼1 spike per 2 ms or 50 sample points), a similar response to PEDOT‐PSS‐ and PEDOT‐DBSA‐modified electrodes.^35^ As the probe was inserted and retracted from the IC, the SNR varied, with no obvious difference between responses inside or outside the IC, with one of the best‐resolved responses for PEDOT‐DS being displayed in Figure 4A. In contrast, the spike count difference had a sharp transition when entering the IC and was far more stable (Figure 4B). Subsequently, the spike count difference was used to define when an electrode was in the IC (45% of the maximum spike count difference produced the most uniform response across electrodes). While all probes showed an increase in spike count difference during experimentation, it was found that one PEDOT‐DS electrode only grazed the IC, resulting in insufficient data for statistical analysis, and was subsequently excluded.

**TABLE 1:** Average (Ave), standard deviation, and coefficient of variation (CV) of RMS_bkgd_, signal‐to‐noise ratio, and mean during stimulation spike count.

Polymer coating	RMS_bkgd_ (µV)			Signal‐to‐noise ratio			Mean during stimulation spike count		
	Ave	SD	CV	Ave	SD	CV	Ave	SD	CV
15‐s PEDOT‐CS	9.4	1.1	0.12	3.50	0.36	0.10	20.1	2.2	0.11
30‐s PEDOT‐CS	11.2	2.7	0.24	3.25	0.61	0.19	20.4	2.0	0.10
45‐s PEDOT‐CS	10.8	1.4	0.13	3.29	0.47	0.14	21.1	1.2	0.06
60‐s PEDOT‐CS	10.4	4.1	0.40	3.51	0.94	0.27	19.9	2.9	0.15
45‐s PEDOT‐pTs	14.5	5.5	0.38	2.93	0.79	0.27	20.4	1.9	0.09
Uncoated	20.2	11.1	0.55	1.73	0.30	0.17	8.5	3.7	0.43
15‐s PEDOT‐DS	8.7	1.2	0.14	3.96	0.95	0.24	22.4	0.8	0.04
30‐s PEDOT‐DS	10.5	3.0	0.28	3.23	0.57	0.18	22.8	0.7	0.03
45‐s PEDOT‐DS	9.5	1.7	0.18	3.28	0.72	0.22	22.4	0.4	0.02
60‐s PEDOT‐DS	9.6	4.7	0.49	3.51	1.29	0.37	22.2	0.5	0.02
45‐s PEDOT‐pTs	13.5	6.5	0.48	3.19	1.20	0.38	22.4	0.8	0.04
Uncoated	19.2	11.1	0.58	1.53	0.12	0.08	4.9	2.6	0.53

As the probes were inserted and retracted within the IC, the RMS_bkgd_, RMS_stim_, SNR, and spike count varied. By averaging the response of individual electrodes at each position in the IC, the biological noise (associated with varying numbers, types, and positions of active neurons near the electrode) was significantly reduced. Nevertheless, the coefficient of variation of the RMS and SNR remained larger than for the spike count (Table 1). Furthermore, the categorization of an electrode as having a good SNR (defined as being >4^38^) was highly impacted by biological noise. The SNR of all electrodes, after adjusting for biological noise, were classified as low–medium, regardless of coating. If instead biological noise was ignored and the SNR had been calculated from the maximum response at any point in the IC, the majority of PEDOT‐modified electrodes would have been classified as good (exceptional even), with uncoated electrodes remaining as low. Subsequently, SNR measurements that have not been corrected for biological noise may impact the accuracy of the measurements and limit the ability to compare the response from other studies and electrode materials.

**FIGURE 5:**
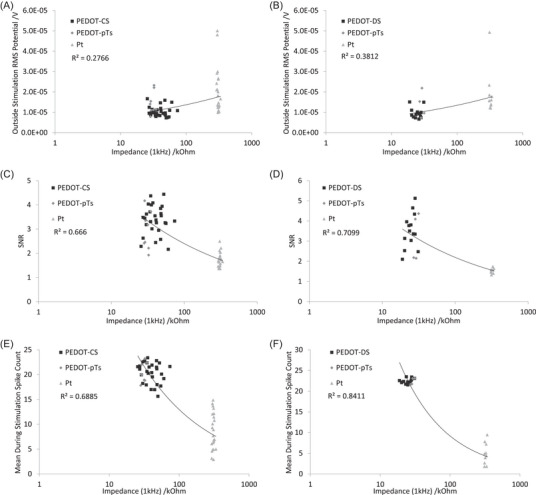
Impedance at 1 kHz at (A, C, E) PEDOT‐CS‐ and (B, D, F) PEDOT‐DS‐modified electrodes versus (A, B) RMS_bkgd_, (C, D) signal‐to‐noise ratio (SNR), and (E, F) mean during stimulation spike count. The fitted trendlines are power curves.

EIS measurements vary with the electrochemical setup, but in a simple RC circuit, the high frequencies are dominated by the solution properties, while low frequencies are dominated by the electrode properties. Regardless of the electrochemical system, it is common for the impedance of neural electrodes to only be assessed at 1 kHz. This study found an increase in RMS_bkgd_ and a decrease in SNR and spike count with increasing impedance at 1 kHz (Figure 5). The relevance of impedance to electrophysiological response is usually attributed to thermal noise. A decrease in thermal noise reduces the RMS_bkgd_, and subsequently increases SNR and spike count. The equation for thermal noise is

(3) $$V_{rms}^{th}=\sqrt{4k_{b}TZ^{\prime }\Delta f}\;,$$

where *Z′* is the real part of the impedance, *k_b_* is Boltzmann's constant, *T* is the absolute temperature, and Δ *f* is the measuring bandwidth.^43^ In an RC circuit, the equation can be simplified to

(4) $$V_{rms}^{th}=\sqrt{\frac{k_{b}T}{C}}\;,$$

where *C* is the electrode capacitance. Subsequently, a metal electrode in contact with a simple electrolyte, which is modeled as an RC circuit with decreasing impedance versus frequency function, may allow measurement of an electrode's thermal noise by measuring impedance at 1 kHz (or any other frequency below the Maxwell–Wagner relaxation frequency).^35^

However, the modification of an electrode with novel materials may remove the decreasing dependence of impedance versus frequency (lowering the Maxwell–Wagner relaxation frequency), altering its equivalent circuit and thermal noise. The choice of impedance frequency may then affect the ability to assess an electrode's thermal noise. As seen with the PEDOT‐modified electrodes, the impedance at 1 kHz can fall on or above the Maxwell–Wagner relaxation frequency, resulting in a response affected by multiple parameters, limiting its utility and reliability for predicting electrophysiological performance.

**FIGURE 6:**
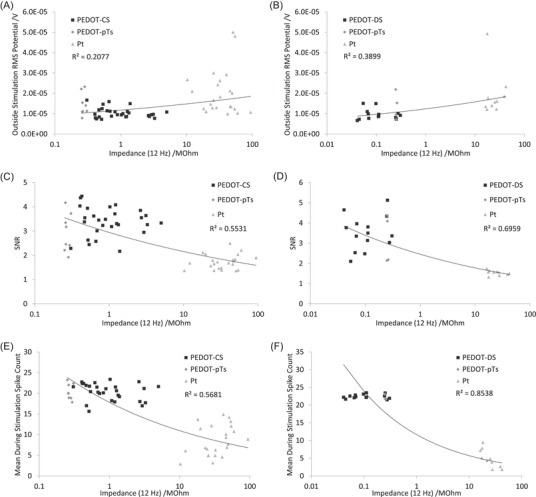
Impedance at 12 Hz at (A, C, E) PEDOT‐CS‐ and (B, D, F) PEDOT‐DS‐modified electrodes versus (A, B) RMS_bkgd_, (C, D) signal‐to‐noise ratio (SNR), and (E, F) mean during stimulation spike count. The fitted trendlines are power curves.

To better assess the relationship between electrophysiological behavior and electrode properties, the electrophysiological response was plotted against impedance at 12 Hz (Figure 6). There were similar trends in the electrophysiological response versus impedance at 12 Hz and 1 kHz. This similarity was due to a strong correlation between impedance at 12 Hz and 1 kHz in these well‐controlled experiments. However, there were slight differences in the correlation coefficient when using the two impedance frequencies, particularly the PEDOT‐CS electrophysiology being less correlated with impedance at 12 Hz (Figures 5 and 6). This begs the question of where the increased variability is arising. For PEDOT‐CS with 15‐ to 60‐s deposition times, the coefficient of variation in impedance at 1 kHz ranged from 0.25 to 0.45, and at 12 Hz from 0.17 to 0.43;^34^ for PEDOT‐DS with 15‐ to 60‐s deposition times, the coefficient of variation of impedance at 1 kHz ranged from 0.05 to 0.16, and at 12 Hz from 0.12 to 0.17;^32^ while PEDOT‐pTs with a 45‐s deposition time had a coefficient of variation of impedance at 1 kHz of 0.08 and at 12 Hz of 0.07.^34^ Subsequently, the coefficient of variation of PEDOT‐CS is roughly double that for PEDOT‐DS and four times larger than PEDOT‐pTs. However, the coefficient of variation was similar at the two different impedance frequencies for individual coatings. In contrast, uncoated platinum had a lower coefficient of variation in impedance at 1 kHz (0.03) compared to the coated electrodes, but a substantially larger coefficient of variation at 12 Hz (0.50), with platinum electrodes on the PEDOT‐CS probes being more variable than on the PEDOT‐DS probes. And while there were differences in electrode area between each coating, the coefficient of variation in area at different deposition times was substantially lower than for all impedance measurements, suggesting variability in electrode area is not impacting the correlation coefficients (see Refs. 30 and 33 for details). This indicates the decreased correlation coefficient for PEDOT‐CS at 12 Hz is in part related to the increased variability in the uncoated platinum impedance (we do not speculate on the cause of this variability).

The impedance at 12 Hz is a function of electrode capacitance, charge transfer resistance, and Warburg impedance. The typical reporting of total impedance of bionic electrodes and its relationship with thermal noise makes the assumption that each of these distinct electrochemical properties have an equivalent impact on electrophysiological performance. It is possible to plot the electrophysiological response against individual fitted equivalent circuit terms to gain a greater understanding of which parameters control the electrophysiological behavior. However, there is an error in the fitting process that can limit the utility of this approach. A greater issue is that each electrode material in this set of experiments required a different equivalent circuit, resulting in different fitting parameters and preventing the plotting and correlation of the fitted electrochemical parameters and electrophysiological response.

To bypass the limitations of the EIS technique, it is possible to obtain various electrode parameters via voltammetric techniques. It was previously shown that the CSC and charge density had no relationship to the electrophysiological recordings, indicating Faradaic processes do not impact the recordings.^27^ It is also possible to use voltammetry to measure the electrode area, enabling three different measures of the electrode area, a two‐dimensional geometric area determined by microscopy, and two electroactive areas determined by a linear or radial diffusion profile of Ru(NH_3_)_6_^3+^.^10^, ^29^ The linear diffusion electroactive area is more sensitive to surface roughness. The relationship of impedance to electrode area depends on the equivalent circuit, and in the case of the simplified Randles circuit (Figure 2D), it has the form $Z=a/A^{2}+b/A+c$.^31^ Trendlines of this form also produced strong correlations of impedance to electrode area for PEDOT‐DS and PEDOT‐CS‐modified electrodes^32^, ^34^ and were subsequently applied to plots of electrode area versus electrophysiological response.

**FIGURE 7:**
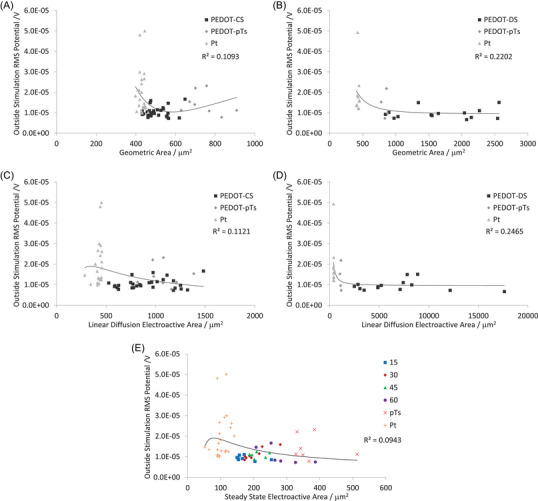
RMS_bkgd_ of (A, C, E) PEDOT‐CS‐ and (B, D) PEDOT‐DS‐modified electrodes versus (A, B) geometric area, (C, D) linear diffusion electroactive area, and (E) steady state electroactive area. The fitted trendlines are $R=a/A^{2}+b/A+c$.

**FIGURE 8:**
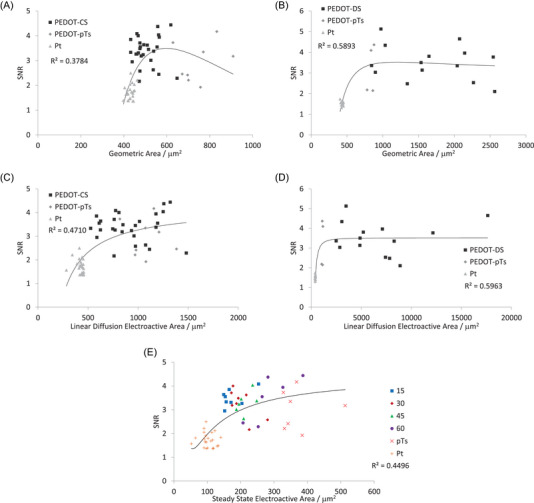
Signal‐to‐noise ratio (SNR) of (A, C, E) PEDOT‐CS‐ and (B, D) PEDOT‐DS‐modified electrodes versus (A, B) geometric area, (C, D) linear diffusion electroactive area, and (E) steady state electroactive area. The fitted trendlines are $R=a/A^{2}+b/A+c$.

**FIGURE 9:**
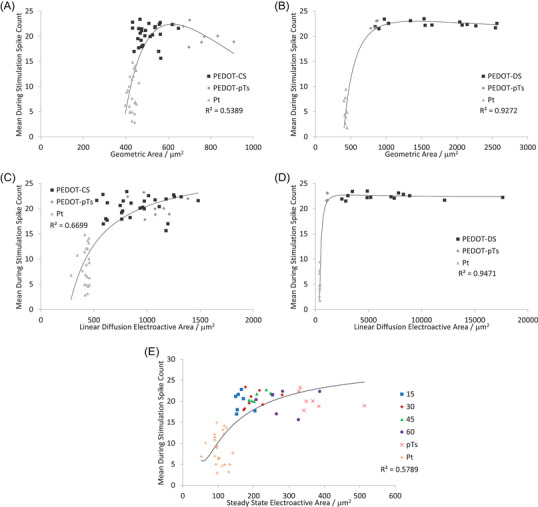
Mean during stimulation spike count of (A, C, E) PEDOT‐CS‐ and (B, D) PEDOT‐DS‐modified electrodes versus (A, B) geometric area, (C, D) linear diffusion electroactive area, and (E) steady state electroactive area. The fitted trendlines are $R=a/A^{2}+b/A+c$.

The RMS_bkgd_ was poorly correlated with any measure of electrode area (Figure 7). There were moderate correlations between SNR and electrode area, with a linear diffusion electroactive area generating a slightly higher correlation than other area measurements, and PEDOT‐DS having higher correlations than PEDOT‐CS (Figure 8). Very strong correlations were seen between spike count and electrode area, again with linear diffusion electroactive area generating a higher correlation than other area measurements, and PEDOT‐DS having a stronger correlation than PEDOT‐CS (Figure 9). We note that the trendline is being applied across all electrodes (equivalent circuits), including PEDOT‐pTs and uncoated platinum. Furthermore, the relationship between electrophysiology and electrode area may not be the same as between area and impedance; however, the trendline was able to produce very strong correlations. The development of more advanced electrochemical models of the electrode−tissue interface is required to generate a theoretical‐based trendline for the electrode area versus electrophysiological response, as discussed below, and will be the focus of future publications.

The difference in the correlations of electrophysiological response between PEDOT‐CS and PEDOT‐DS was partly due to the probes being more variable (as noted above, the coefficient of variation in PEDOT‐CS impedance was nearly double PEDOT‐DS). Separation of the electrochemical performance of each PEDOT‐CS probe revealed one had a substantially higher coefficient of variation than the other (again, we avoid speculation on the cause of this variability). Two animals were also used to assess PEDOT‐CS, while only one was available for PEDOT‐DS. Separation of the data from each PEDOT‐CS probe resulted in the correlation coefficients of all electrophysiology curves versus impedance or electroactive area curves increasing by 0.01 for one probe, and 0.1 for the second probe. This demonstrates the difficulty in assigning levels of noise to the different probes or animals (thermal noise or biological noise). Electroanalysis of individual probes must be performed to determine their thermal noise and variability, but it is not yet clear if a correction of the electrophysiological response using the electrochemical performance is possible. The current experimental approach can reduce biological noise within one animal. It may be possible to reduce biological noise across different animals by normalizing the electrophysiological data from each animal, for example, to a maximum RMS, SNR, or spike count. However, a normalization approach would only apply to the specific set of experiments being analyzed, preventing its use across other experiments. Therefore, we recommend the reporting of absolute electrophysiological responses rather than normalized values.

The variability between animals and probes raises more fundamental concerns around the need for standardizing electrophysiology experiments. Without some form of standardization, it is not possible to compare different electrodes, animals, experimental protocols, or noise processes. An investigation of standardization in electrophysiology will be published in a forthcoming article. Indeed, other groups are also attempting to standardize the reporting requirements for bionic devices.^44^

The trends seen between the electrophysiological and electrochemical performance revealed an increase in electrode area reduced the total impedance, which can reduce thermal noise and increase SNR and spike count. However, a larger electrode also increases the neural population within the recording distance. The decrease in RMS_bkgd_ with increasing electrode area was smaller than the increase in RMS_stim_ and SNR. RMS_bkgd_ was also poorly correlated with electrode area, while SNR and spike count were moderately to highly correlated. This indicates that the decrease in thermal noise with increasing electrode area is a minor factor, with increased neural population within recording distance being more important. This key finding will be further investigated in a forthcoming article using iridium oxide electrodes.

Computational modeling of electrophysiological recording has been reported previously, with peak‐to‐peak amplitude of neural spikes decreasing with increased electrode area and electrode‐neuron distance.^45^ Modeling has also shown a similar magnitude decrease in thermal noise with increased electrode area, as was seen in the current experimental study.^46^ However, in contradiction with the experimental evidence presented in this article, the model also predicted a decrease in SNR with increasing electrode area. One reason for this discrepancy in SNR response may be that the model failed to include a sufficient neural population for the increased volume of tissue accessible with larger electrode areas. Of more fundamental concern is that these models are based on cable theory, where the electrochemical properties of tissue are assumed to be ohmic. This unrealistic model assumes an electric field drops off with a 1/*r* ^2^ profile rather than exponentially. Subsequently, the volume of tissue in the recording distance of an electrode would be substantially smaller than modeled. These types of models also ignore other chemical, physical, and thermodynamic processes that may impact electrophysiological recordings, including variations in dielectric constant associated with changing ionic concentrations, temperature changes, and cell membrane movement during action potential propagation. This highlights the limited theoretical understanding of charge transport in electrophysiology and the need to expand the application of electroanalytical techniques to neuroscience.

We note that the present work investigated the acute electrophysiological performance of the electrodes. There was no electrode damage visible via optical microscopy from the implantation process. The electrodes were only used for neural recording experiments, so issues related to cracking or delamination of the conducting polymer from the underlying metal electrode, associated with current injection during neural stimulation, were avoided.^25^ During the short implantation period, protein fouling on the electrode surface will occur, but there will not have been sufficient time for other foreign body reaction processes to occur. Subsequently, histological analysis and biocompatibility tests were of limited value. Protein fouling results in partial blocking of the electrode, which can be measured by fast cyclic voltammetry of Ru(NH_3_)_6_^3+^.^42^ Unfortunately, the voltammetry of the electrodes after implantation was significantly distorted, preventing electrochemical modeling and calculation of a blocked electrode area.

During chronic implantation, assuming the electrode is unaffected, a foreign body reaction may occur, leading to encapsulation of the electrode by glial tissue and increased electrode‐neuron distances. This should have a minimal impact on RMS_bkgd_ but reduce RMS_stim_, SNR, and spike count. Improved theoretical modeling, as described above, would also help determine the neural population being recorded in these conditions. We further note that, in chronic studies, a two‐electrode configuration is often used with a platinum or steel counter electrode, which are polarizable quasi‐electrodes with different DC potentials. However, electrophysiological recordings measure local changes in potential with very low current flux and the measurements are high pass filtered, largely removing the effect of different DC potentials between electrodes; furthermore, the counter electrode is typically significantly larger than the working electrode, so that charge transport kinetics are controlled by the working electrode and any current flow has minimal effect on the counter electrode potential.^47^ Subsequently, minimal impact from different counter electrode materials or configurations is expected on the electrophysiological response.

## CONCLUSION

Neural recording electrodes were assessed by electrochemical and electrophysiological methods to determine the relationship between the two techniques. Uncoated and conducting polymer–coated electrodes provide a range of electrochemical responses that help determine their impact on the electrophysiological performance. An increased electrode area lowered the total impedance at low frequencies (below the Maxwell–Wagner relation frequency). This led to a small decrease in thermal or electrochemical voltage noise. A larger electrode also increased the volume of neural tissue within the recording range, leading to an increase in SNR and spike count. Previous computational modeling has shown that the peak‐to‐peak amplitude of a spike decreases with increasing electrode area.^45^ There appeared to be a plateauing of SNR and spike count at very large electrode areas, indicating a balance between decreasing thermal noise and a larger population of neurons being measured with decreasing signal amplitude. This balance may depend on the particular neural structure, type, size, firing rate, and other electrode properties and requires the development of new electrochemical models.

Correlations between electrochemical and electrophysiological recording demonstrated multiple noise sources, including thermal noise and biological noise—both within an animal and between different animals—requiring methods to independently assess and correct each noise source. A surgical procedure measuring electrophysiological response from multiple locations within the IC significantly reduced biological noise within one animal, but not between animals. In particular, there was a relatively large variability in the electrochemical performance of the uncoated platinum electrodes on one probe, which led to variability in its thermal noise and electrophysiological response, impacting the correlations of PEDOT‐CS‐modified electrodes. Nevertheless, very strong correlations were obtained between a linear diffusion electroactive area and electrophysiological response, with spike count being more accurate than SNR.

The correlation of electrochemical and electrophysiological performance indicates that thermal noise has a relatively small impact on electrophysiological recording, compared to the increased neural population being measured. As a result, impedance is mainly useful as an indirect measure of electrode area, in which case, the measurement and analysis of EIS must be performed appropriately. Furthermore, computational modeling of electrophysiology needs to be improved through the application of realistic electrochemical theory. The development of standard electrodes and techniques would assist in the development and comparison of this new theory, bionic devices, and their electrophysiological performance.

## AUTHOR CONTRIBUTIONS

ARH: Conceptualization, methodology, investigation, formal analysis, and writing—original draft. BJA: Investigation and writing—review and editing. AGP: Resources.

## COMPETING INTERESTS

The authors declare that they have no known competing financial interests or personal relationships that could have appeared to influence the work reported in this paper.

## PEER REVIEW

The peer review history for this article is available at: https://publons.com/publon/10.1111/nyas.15371.

## Data Availability

All data generated or analyzed during this study are included in this published article.

## Footnotes

^a^
To our knowledge, the first reference to the 1 kHz impedance “myth” was incorrectly citing a textbook. The only potentially relevant mention to 1 kHz we found in the textbook was to imagine an action potential propagated in 1 ms. This propagation velocity has no impact on the ability of an electrode to measure the potential at the electrode−tissue interface.
